# Increasing prevalence of cirrhosis among insured adults in the United States, 2012–2018

**DOI:** 10.1371/journal.pone.0298887

**Published:** 2024-02-26

**Authors:** Daniela P. Ladner, Michael Gmeiner, Bima J. Hasjim, Nikhilesh Mazumder, Raymond Kang, Emily Parker, John Stephen, Praneet Polineni, Anna Chorniy, Lihui Zhao, Lisa B. VanWagner, Ronald T. Ackermann, Charles F. Manski

**Affiliations:** 1 Northwestern University Transplant Outcomes Research Collaborative (NUTORC), Comprehensive Transplant Center (CTC), Northwestern University, Chicago, IL, United States of America; 2 Department of Surgery, Division of Organ Transplantation, Northwestern University, Chicago, IL, United States of America; 3 Department of Economics, London School of Economics, London, United Kingdom; 4 Department of Medicine, Division of Hepatology, University of Michigan, Ann Arbor, MI, United States of America; 5 Institute for Public Health and Medicine (IPHAM), Northwestern University, Chicago, IL, United States of America; 6 UnitedHealth Group, United States of America; 7 Department of Preventive Medicine, Northwestern University, Chicago, IL, United States of America; 8 Department of Medical Social Sciences and Buehler Center for Health Policy and Economics, Northwestern University, Chicago, IL, United States of America; 9 Department of Medicine, Division of Digestive and Liver Diseases, University of Texas Southwestern Medical Center, Dallas, TX, United States of America; 10 Department of Economics and Institute for Policy Research, Northwestern University, Evanston, IL, United States of America; Institute for Clinical and Experimental Medicine, CZECH REPUBLIC

## Abstract

**Background:**

Liver cirrhosis is a chronic disease that is known as a “silent killer” and its true prevalence is difficult to describe. It is imperative to accurately characterize the prevalence of cirrhosis because of its increasing healthcare burden.

**Methods:**

In this retrospective cohort study, trends in cirrhosis prevalence were evaluated using administrative data from one of the largest national health insurance providers in the US. (2011–2018). Enrolled adult (≥18-years-old) patients with cirrhosis defined by ICD-9 and ICD-10 were included in the study. The primary outcome measured in the study was the prevalence of cirrhosis 2011–2018.

**Results:**

Among the 371,482 patients with cirrhosis, the mean age was 62.2 (±13.7) years; 53.3% had commercial insurance and 46.4% had Medicare Advantage. The most frequent cirrhosis etiologies were alcohol-related (26.0%), NASH (20.9%) and HCV (20.0%). Mean time of follow-up was 725 (±732.3) days. The observed cirrhosis prevalence was 0.71% in 2018, a 2-fold increase from 2012 (0.34%). The highest prevalence observed was among patients with Medicare Advantage insurance (1.67%) in 2018. Prevalence increased in each US. state, with Southern states having the most rapid rise (2.3-fold). The most significant increases were observed in patients with NASH (3.9-fold) and alcohol-related (2-fold) cirrhosis.

**Conclusion:**

Between 2012–2018, the prevalence of liver cirrhosis doubled among insured patients. Alcohol-related and NASH cirrhosis were the most significant contributors to this increase. Patients living in the South, and those insured by Medicare Advantage also have disproportionately higher prevalence of cirrhosis. Public health interventions are important to mitigate this concerning trajectory of strain to the health system.

## Introduction

The burden of cirrhosis on the healthcare system is projected to increase with the aging of the “baby boom” generation [1]. Usually an indolent process, cirrhosis can progress to hepatocellular carcinoma (HCC) and life-threatening decompensation events at rates of 1–8% and 5–7% per year respectively [2–5]. Cirrhosis-related mortality has risen by 65% over the last decade and management requires vigilant ultrasound screening for HCC, routine esophagogastroduodenoscopies, vaccinations, and regular laboratory monitoring of liver function [4,6–9]. In addition to the concomitant severe polymorbidity in patients with cirrhosis, high rates of hospitalizations and associated costs of care exceed $16.3 billion in 2015 [10–14].

Despite the associated morbidity, mortality, and cost in the United States (US), contemporary estimates of prevalence are not well established and may differ based on methodology [15–18]. US population surveys and database studies relying on cross-sectional methods have varying estimates of cirrhosis prevalence, fluctuating from 0.27%-1.06% [19–23]. Although national databases such as the National Health and Nutrition Examination Survey (NHANES), Surveillance, Epidemiology, and End Results (SEER), and National Inpatient Sample (NIS) are great tools to assess large sample sizes of population health data, they may be prone to self-report and selection bias [24,25]. Smaller studies and those constrained to geographic regions outside of the US may also suffer from generalizability as differing cultural and health care systems exists [26,27]. Gaps in the literature remain for large-scale population health studies in the US.

Specialist care and screening measures are vital to reducing the burden of cirrhosis sequalae. Thus, the primary objective of this study is to ascertain the burden of disease by assessing the prevalence of cirrhosis.

## Materials and methods

### Study design

This was a retrospective, longitudinal cohort study using administrative claims data from one of the largest national insurance companies in the US between 1/1/2011-12/31/2018. This study follows the Strengthening the Reporting of Observational Studies in Epidemiology (STROBE) guidelines [28]. This study was approved by the Northwestern University Institutional Review Board (IRB ID #STU00215803) and also waived the need for patient informed consent due to its retrospective nature. The study data were accessed for research purposes on June 1, 2021. This dataset is de-identified, and authors did not have access to information that could identify individual participants during or after data collection.

### Study participants

The study population comprised of enrolled adults (≥18 years) with (N = 371,492) and without cirrhosis (N = 50,045,769) from a population of 50,417,251 enrolled adult patients in 2011–2018. Cirrhosis diagnosis was defined as having at least one of the previously published International Classification of Diseases, 9th Revision (ICD-9), International Statistical Classification of Diseases and Related Health Problems, Tenth Revision (ICD-10) data and medication prescription (**S1 and S2 Tables**) [29–33]. The first appearance of a cirrhosis code was considered the index date. Diagnosis codes for advanced fibrosis, acute liver failure, or acute hepatitis were not used unless they had been validated previously by the literature as part of the definition for cirrhosis [29–33]. The cohort observation period started 1/1/2011 to allow for 12 months of time to observe medical events and relevant covariates of the cohort. Patients were censored after liver transplant, discontinuation of insurance coverage, or at the end of the observation period (12/31/2018).

### Covariates

Demographic data were captured at the index date and included age, sex, and insurance. There were less than 1% of missing data for age and sex. Patients were either enrolled in commercial, Medicare Advantage plans, or both. Medicare Advantage is a health insurance plan that offers Medicare benefits to eligible patients (e.g., ≥65 years, certain persons with disabilities) and is provided through commercial-sector health insurance companies. Race was excluded from our report because it relied on a combination of self-reporting and an imputation algorithm using geographic variables and surname analyses which decreases the validity of the information.

Diagnosis, cirrhosis etiology, and clinical complications associated with the patient during the study period were defined using all available ICD-9/ICD-10 (1^st^-25^th^ diagnoses) and/or CPT codes used in previously published literature (**S3 Table**) that yield a positive predictive value of 75–97% [29–33]. Etiologies included alcohol-related, non-alcoholic steatohepatitis (NASH), hepatitis C virus (HCV), biliary cirrhosis (e.g., primary sclerosing cholangitis, primary biliary cirrhosis), hepatitis B virus (HBV), cardiac cirrhosis, autoimmune hepatitis, and genetic. Rare etiologies were summarized as ‘Other’ and included cirrhosis with no ascertainably associated etiology. NASH was defined according to published guidance on the use of ICD-10 code or absence of another etiologic diagnosis but having at least one of the following diagnoses: obesity, diabetes, or hypertension [34–36]. Portal hypertension and decompensated cirrhosis were defined as the occurrence of any of the following complications at any point during the observation period: hepatic encephalopathy (HE), ascites, spontaneous bacterial peritonitis (SBP), variceal bleed (VB), hepatorenal syndrome (HRS) and/or hepatopulmonary syndrome (HPS). Patients were classified as compensated if they did not fill any of the prescribed medications or have diagnosis codes for decompensation (**S2 Table**).

The Model for End-stage Liver Disease with Sodium (MELD-Na), the Elixhauser Index and each comorbidity were calculated using previously published measures [37,38]. The MELD-Na score was calculated with the four components of lab data when they were present within 90 days of each other. If multiple scores were available, the highest value was used. Only 57% (N = 210,853) of patients with cirrhosis in the cohort had lab values available to calculate a MELD-Na. LOINC codes for labs are listed in **S4A Table** and dialysis codes in **S4B Table**.

Follow-up was defined as days from the time of first observed cirrhosis diagnosis to the final date of enrollment (12/31/2018) or patient censoring. For patients with decompensated cirrhosis, follow-up was calculated from the number of days between decompensation diagnosis until final date of enrollment or patient censoring.

### Statistical analyses

Categorical variables were expressed in percentage and odds ratio (OR) with respective 95% confidence intervals (CI). Annual prevalence was calculated as patients with cirrhosis divided by all patients enrolled as of July 15 each year for 2012–2018 and presented as a percentage. Patients who joined the cohort with a diagnosis of cirrhosis but did not have a clinic visit during their first year of enrollment, were not captured in the cirrhosis cohort until the following year since the follow-up visit for a cirrhosis-related issue occurs within 2 years for most patient-years (95.4%). Further details on calculation of prevalence are available in **S1 Methods**.

Mann-Whitney-U and chi-square analysis were used to analyze categorical and continuous variables. A multivariable logistic regression analysis on the risk of cirrhosis diagnosis was conducted to identify specific risk factors of interest adjusted for age, gender, insurance, and region of residence in the US. Maps were generated using the Maptile command in Stata and did not require any copyrighted materials [39]. States with ≤70 patients with cirrhosis for a specific year were excluded from analyses to ensure patient anonymity. All statistical analyses were performed with Stata 14.1MP [39].

## Results

Between 2011–2018, the cohort included 50,417,251 enrolled patients among whom 371,492 patients had cirrhosis. The mean (±SD) age was 62.2-years-old (±13.7-years), with 225,089 (60.6%) of the patients ≥60-years-old, 168,021 (45.2%) female, 198,058 (53.3%) having commercial insurance, 172,183 (46.4%) having Medicare Advantage insurance, and 1,251 (0.3%) carrying both. The most frequent etiologies of cirrhosis were alcohol-related 645 (26.0%), NASH 77,716 (20.9%), HCV 74,433 (20.0%), biliary 25,882 (7.0%), HBV 19,120 (5.2%), and Other 110,981 (29.9%). A transjugular intrahepatic portosystemic shunt (TIPS) was performed in 3,121 (0.8%) of all patients with cirrhosis. The mean Elixhauser Index score for the cohort was 3.03 (±2.92) (**Table 1**).

**Table 1:** Cohort demographics by compensated and decompensated cirrhosis state.

	Total (N = 371,492)	Compensated (N = 216,905)	Decompensated (N = 154,587)	p-values
Age; mean (±SD)	62.2 (13.7)	62.0 (13.7)	62.4 (13.6)	
18–39; N (%)	23,853 (6.4%)	14,411 (6.64%)	9,442 (6.11%)	<0.001
40–49; N (%)	35,815 (9.6%)	21,339 (9.84%)	14,476 (9.36%)	<0.001
50–59; N (%)	86,725 (23.4%)	49,899 (23.01%)	36,826 (23.82%)	<0.001
60–69; N (%)	115,054 (31.0%)	68,806 (31.72%)	46,446 (30.05%)	<0.001
>70; N (%)	110,035 (29.6%)	62,648 (28.88%)	47,387 (30.65%)	<0.001
Female; N (%)	168,021 (45.2%)	101,736 (46.90%)	66,285 (42.88%)	<0.001
Insurance				
Commercial; N (%)	198,058 (53.3%)	116,544 (53.73%)	81,514 (52.73%)	<0.001
Medicare; N (%)	172,183 (46.4%)	99,643 (45.95%)	72,540 (46.93%)	<0.001
Both; N (%)	1,251 (0.3%)	718 (0.33%)	533 (0.34%)	0.475
Etiology				
ETOH; N (%)	96,645 (26.0%)	39,076 (18.02%)	57,470 (37.18%)	<0.001
NASH; N (%)	77,716 (20.9%)	44,649 (20.58%)	33,067 (21.39%)	<0.001
HCV; N (%)	74,433 (20.0%)	40,167 (18.52%)	34,266 (22.17%)	<0.001
Biliary; N (%)	25,882 (7.0%)	18,282 (8.43%)	7,600 (4.92%)	<0.001
HBV; N (%)	19,120 (5.2%)	9,394 (4.33%)	9,726 (6.29%)	<0.001
Cardiac; N (%)	10,020 (2.7%)	6,837 (3.15%)	3,183 (2.06%)	<0.001
Autoimmune; N (%)	8,944 (2.4%)	5,676 (2.62%)	3,268 (2.11%)	<0.001
Genetic; N (%)	7,423 (2.0%)	4,667 (2.15%)	2,789 (1.80%)	<0.001
Other; N (%)	110,981(29.9%)	73,264 (33.78%)	37,717 (24.40%)	<0.001
Compensated; N (%)	216,905 (58.4%)	216,905 (100.00%)		
Compensated with portal hypertension; N (%)	112,019 (30.15%)	45,609 (21.03%)	66,410 (42.96%)	<0.001
Decompensation Events; N (%)	154,587 (41.3%)		154,587 (100%)	<0.001
Ascites; N (%)	111,967 (30.1%)		111,967 (72.43%)	
HE; N (%)	50,548 (13.6%)		50,548 (32.70%)	
VB; N (%)	30,048 (8.1%)		30,048 (19.44%)	
HRS; N (%)	13,415 (3.6%)		13,415 (8.68%)	
SBP; N (%)	11,832 (3.2%)		11,832 (7.65%)	
HPS; N (%)	529 (0.1%)		529 (0.34%)	
>1 decompensated Complication; N (%)	47,162 (12.7%)		47,162 (30.51%)	
HCC; N (%)	42,644 (11.5%)	23,727 (10.94%)	18,917 (12.24%)	<0.001
MELD-Na; mean [±SD]	12.5 (7.1)	10.6 (6.0)	14.6 (7.6)	<0.001
Elixhauser Index; mean (±SD)	3.03 (2.92)	2.19 (2.31)	4.19 (3.27)	<0.001
TIPS; N, (%)	3,121 (0.8%)	128 (0.06%)	2,993 (1.94%)	<0.001
Follow-up days; mean (±SD)	725 (732)	696 (720)	645 (702)	<0.001

CI = 95% confidence interval, ETOH = alcohol use disorder, HCC: Hepatocellular Carcinoma, HBV = hepatitis B virus, HCV = hepatitis C virus, HE = Hepatic encephalopathy, HPS = hepatopulmonary syndrome, HRS = hepatorenal syndrome, IQR = interquartile range, MELD-Na = Model for End-stage Liver Disease with Sodium, N = Number of patients, NASH = Non-alcoholic steatohepatitis, OR = Odd Ratio, Other = None of the listed complications; PHTN = portal hypertension, SD = standard deviation, SBP = spontaneous bacterial peritonitis, TIPS = transjugular intrahepatic portosystemic shunt, VB = variceal bleeding.

Compensated and decompensated cirrhosis: There were 216,905 (58.4%) patients who had compensated cirrhosis, while 154,587 (41.6%) patients either initially had or developed decompensated cirrhosis. Among those with decompensated cirrhosis, 111,967 (72.4%) had ascites, 50,548 (32.7%) with HE, 30,048 (19.4%) with VB, 13,415 (8.7%) with HRS, 11,832 (7.7%) with SBP, 529 (0.3%) with HPS, and 47,162 (30.5%) presented with more than one decompensating complication. The mean MELD-Na score was 12.5 (±7.1) for compensated patients 10.6 (±6.0) and 14.6 (±7.6) for decompensated patients (p<0.001, **Table 1**).

**Fig 1:**
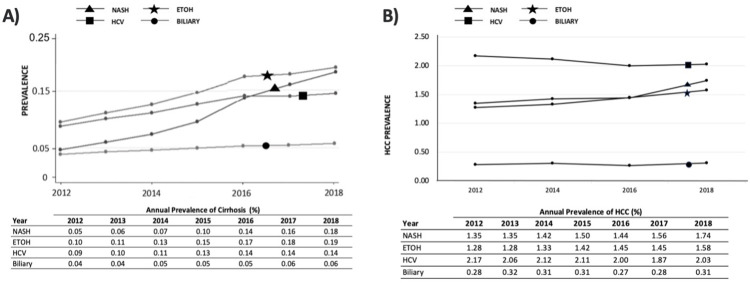
Prevalence of cirrhosis and HCC stratified by etiology. ETOH = alcohol-related, HCV = hepatitis C virus, NASH = non-alcoholic steatohepatitis.

Cirrhosis Prevalence: The prevalence of cirrhosis and HCC have been consistently increasing since 2012 (**Fig 1A and 1B**). The overall unadjusted prevalence of cirrhosis was 0.71% in 2018, a 2.1-fold increase from 0.34% in 2012 (p<0.001). By etiology, the prevalence increased 0.10% to 0.19% for alcohol-related (p<0.001), 0.09% to 0.15% for HCV (p<0.001), and 0.04% to 0.06% for biliary cirrhosis (p<0.001) (**Table 2, Fig 1A**). By 2018, HCC affected 7.37% of all patients with cirrhosis: 2.03% in HCV cirrhosis, 1.74% in NASH, 1.58% alcohol-related, and 0.31% in biliary cirrhosis. From 2012–2018, patients with NASH cirrhosis had the largest increase in HCC prevalence (129.4%) while HCV cirrhosis had a decrease in HCC prevalence (6.7%) (**Fig 1B**).

**Table 2:** Cirrhosis prevalence of patients enrolled compared by year.

Year	Patients enrolled	Cirrhosis	Annual Prevalence (%)										
			All	Insurance		Etiology				Regions of the US			
				Medicare Advantage	Commercial	NASH	EtOH	HCV	Biliary	Northeast	Midwest	South	West
2012	19,740,634	67,025	0.34	0.88	0.25	0.05	0.10	0.09	0.04	0.30	0.21	0.29	0.31
2013	20,653,786	84,204	0.41	1.07	0.30	0.06	0.11	0.10	0.04	0.35	0.26	0.36	0.37
2014	19,618,517	89,954	0.46	1.21	0.32	0.07	0.13	0.11	0.05	0.40	0.29	0.40	0.43
2015	19,036,572	99,018	0.52	1.35	0.34	0.10	0.15	0.13	0.05	0.45	0.32	0.47	0.50
2016	20,202,190	122,385	0.61	1.52	0.37	0.14	0.17	0.14	0.05	0.49	0.38	0.56	0.58
2017	21,771,537	140,755	0.65	1.60	0.37	0.16	0.18	0.14	0.06	0.54	0.41	0.61	0.61
2018	21,110,112	149,216	0.71	1.67	0.38	0.18	0.19	0.15	0.06	0.58	0.45	0.67	0.68
**% Increase between 2012–18**			208.2%	189.8%	152.0%	387.2%	200.0%	164.8%	148.7%	192.5%	210.0%	229.2%	218.9%
**p-value**			<0.001	<0.001	0.001	<0.001	<0.001	<0.001	<0.001	0.001	0.002	0.003	0.003

ETOH = alcohol-related, HCV = hepatitis C virus, NASH = non-alcoholic steatohepatitis, US = United States.

**Fig 2:**
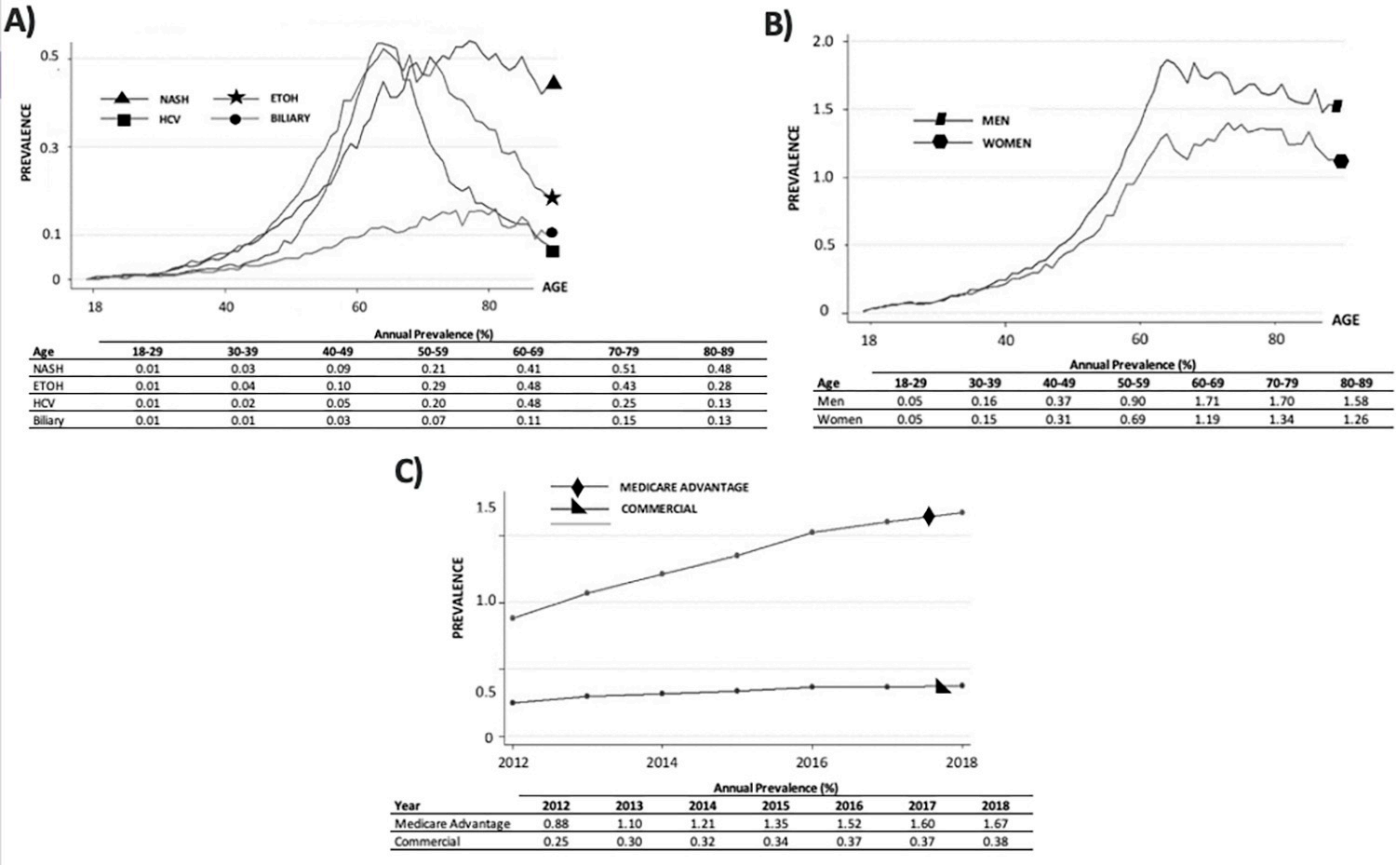
Prevalence of cirrhosis, and its etiologies, by age, gender, and insurance over time. ETOH = alcohol-related, HCV = hepatitis C virus, NASH = non-alcoholic steatohepatitis.

**Fig 3:**
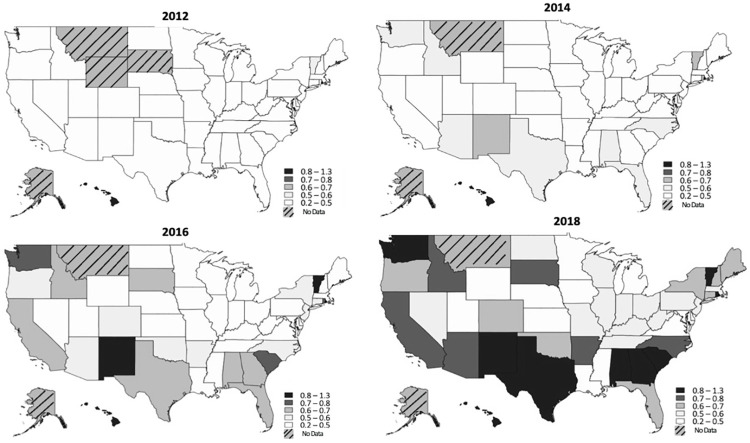
US prevalence of insured Americans with cirrhosis between 2012–2018. Prevalence of cirrhosis of insured Americans from 2012 to 2018 by state have progressively increased. “No data” refers to states with ≤70 patients with cirrhosis for the year.

The prevalence of NASH and male patients with cirrhosis increased disproportionately more with age compared to other etiologies of cirrhosis and female gender respectively (**Fig 2A and 2B**). The fastest increase in prevalence was for NASH (0.05% to 0.18%, p<0.001), with a disproportionate 1-year increase between 2015–2016 by 42.7%. The highest cirrhosis prevalence was observed in patients with Medicare Advantage insurance (1.67% in 2018) compared to commercial insurance (0.38% in 2018) (**Table 2, Fig 2C**). In every US state, prevalence increased each year from 2012–2018 (**Fig 3, S5 Table**). The South, West, Midwest, and Northeast saw increases in prevalence by 229.2% (p = 0.003), 218.9% (p = 0.003), 210.0% (p = 0.002), and 192.5% (p = 0.001) respectively (**Table 2**).

Odds of cirrhosis. A multivariable logistic regression analysis assessing the risk of cirrhosis diagnosis was performed adjusting for gender, age, insurance status, and region of residence in the US. Patients with commercial insurance (OR 0.60, CI 0.59–0.60, p<0.001) had lower risk of cirrhosis diagnosis compared to patients with Medicare Advantage. Compared to the Northeast, Patients living in the West (OR 0.92, CI 0.92–0.94, p<0.001) and South (OR 1.07, CI 1.06–1.08, p<0.001) had lower and increased risks of cirrhosis diagnosis respectively (**Table 3**).

**Table 3:** Multivariable logistic regression analysis for risk of cirrhosis.

Risk Factor	OR	95% CI	p-value
Gender			
Female	Reference		
Male	1.44	1.43–1.45	<0.001
Age	1.05	1.05–1.05	<0.001
Insurance status			
Medicare Advantage	Reference		<0.001
Commercial	0.60	0.59–0.60	<0.001
Region of the US			
Northeast	Reference		<0.001
Midwest	0.86	0.85–0.87	<0.001
South	1.07	1.06–1.08	<0.001
West	0.92	0.92–0.94	<0.001

CI = 95% confidence interval, OR = odds ratio, US = United States.

## Discussion

The current study demonstrates the increasing burden of cirrhosis in the US within the past decade. Among a longitudinal cohort of patients enrolled by a large national insurer, cirrhosis prevalence more than doubled over a 6-year period with contemporary overall prevalence of 0.71% in 2018. The most significant increase in prevalence was observed in patients with NASH, while other notable subgroups include alcohol-related etiology (2-fold), patients living in the South (2.3-fold), West (2.2-fold) and those with Medicare Advantage insurance (1.9-fold). This rise in prevalence of cirrhosis, especially of those with decompensated cirrhosis and HCC, suggests an anticipated increase in the public health burden of cirrhosis care [3,14,40].

In this longitudinal cohort of privately insured patients with cirrhosis, the prevalence of cirrhosis was found to be similar to the reported prevalence of 0.27%-1.06% in different cohorts [19–23]. These differences are likely due to variation in study methodology and cohort selection. For example, the smaller prevalence of 0.27% from an NHANES study was limited to patients with available lab values, is subject to survey response bias, and represents an earlier era where known risk factors of cirrhosis were less prevalent [23]. Alternatively, Beste et al., reported a cirrhosis prevalence of 1.06% among a US cohort insured by the Veterans Affairs in 2001–2013 [19]. Although this cohort was also followed longitudinally, the cohort was predominantly male (97.0%), and had comparatively higher comorbidity burdens [19]. Our cohort is most similar to those of Mellinger et al.’s cohort of 115,510,639 patients from the Truven MarketScan Commercial Claims and Encounters database which found that the prevalence among privately insured patients was 0.27% in 2015 [41]. We similarly report a prevalence of 0.34% in 2012 that doubled over time to 0.71% by 2018, emphasizing its growing public health burden.

The rise in cirrhosis prevalence is most driven by the collective increase of alcohol-related and NASH cirrhosis by 2- and nearly 4-fold respectively. The steady growth of alcohol-related cirrhosis parallels the increase in alcohol consumption of persons ≥50-years-old and reports of binge drinking episodes, which have accelerated by 10-fold over the past decade [42–44]. Rates of AUD and emergency department visits involving alcohol consumption have risen to 49.4% and 61.6% respectively [44,45]. These trends are anticipated to worsen due to the impacts of the stay-at-home regulations and social distancing policies during the Coronavirus Disease 2019 (COVID-19) pandemic, that created an environment conducive to the consumption of alcohol [46]. In fact, ALD is now the most common indication for liver transplant listing during the COVID-19 pandemic [46–48]. There is a growing need for public health policies to temper the rise of AUD and support the increasing demand of early liver transplantation protocols [49,50].

The growth of NASH has been attributed in part to the rise of obesity, diabetes, and hypertension [18,51–53]. Among cirrhosis etiologies, NASH experienced the highest rise in prevalence–increasing from 12.5% to 42.9% in our study. In parallel, the prevalence for obesity, diabetes, and hypertension are all anticipated to increase to 50.7%, 21%, and 41.4% respectively by 2030 [54–56]. This has implications among rates of liver transplantation as the annual NASH-related additions to the liver transplant waitlist are anticipated to increase by 55.4% [25]. This persistent rise of metabolic syndrome and its sequelae also reflects the associated increased awareness of NASH [57]. Introducing a novel code for NASH in the ICD-10 codebook on October 1, 2015, will continue to optimize the identification of NASH and fuel future research. This change was unique to NASH and other etiologies of cirrhosis did not have significant changes in their corresponding trajectories. Longitudinal observational cohorts (e.g., TARGET-NASH) will be vital in identifying emerging treatments for NASH, such as statins, and weight loss, in order to slow its rise in prevalence [58–61]. Continued research is warranted to help address the underlying rise of cirrhosis, especially in the post-pandemic era, with NASH and alcohol-related etiologies.

Taken together, although cirrhosis prevalence has collectively increased in the US, differing rates of growth exist between states–particularly among those in the South and West. By 2018, the South and West had nearly a 2.3- and 2.2-fold increase in cirrhosis prevalence respectively. Of the top 10 states with the highest increases in cirrhosis prevalence, 5 states were in the South. Variation among state-level comorbidities and demographics may result in these geographic differences. The highest rates of obesity, diabetes, and other metabolic syndromes are most concentrated in the Southern US [62–64]. This might be further related to poverty since 12 of 15 of the poorest states are from the South and are associated with poor nutrition and liver-related mortality [9,65]. Moreover, the West may be suffering from lower rates of specialty referral due to the sprawling rural landscape [66]. A primary care physician visit in a non-rural area have been associated with a 92% increased probability of specialist referral compared to those in rural areas [67]. Given this, there are regional variations among NASH-related transplantation and future work is necessary to identify high risk patients with disadvantaged socioeconomic status [25]. However, it is interesting that despite high prevalence of diabetes and obesity in the Midwest, there is a lower risk of NASH in these states comparatively. This may be due to the lower liver-focused practices in primary care settings or lower specialist utilization in the region to identify NASH [66]. Further research is required to investigate the associations between cirrhosis prevalence and specialty care referral to affect change in public policy and mitigate the prevalence of cirrhosis.

Besides differences in geographic prevalence of cirrhosis, there were also variation among different insurance enrolment types that highlight individual demographic differences of disease. Prevalence of cirrhosis in Medicare Advantage patients rose at a pace much faster than in those with commercial insurance, increasing by 1.9- and 1.5-fold respectively. Medicare Advantage patients maintained a 38% higher odds of cirrhosis diagnosis compared to those enrolled in commercial insurance. With an aging “baby boom” generation (born in 1946–1964), the Medicare-eligible population is the fastest growing cohort in the US and has already increased by nearly 7-fold for patients with chronic liver disease from 2007–2015 [68,69]. Additionally, cirrhosis-related long-term disability has increased by 30% between 2007–2017 and annual death rates also rose in parallel by 65% [9,70]. Increasing cirrhosis prevalence has implications for individual quality of life as patients with cirrhosis commonly suffer from depression, poor sleep, frailty, and malnutrition [71]. Given the disproportionate rise of cirrhosis prevalence of age-eligible Medicare patients, the observed trajectory is likely to exacerbate healthcare costs and further strain healthcare and societal resources [72,73].

Our findings must be interpreted within the context of the study’s limitations. First, this study is retrospective with patients’ characteristics and covariates defined by diagnosis and procedure codes, incurring the usual caveats to this methodology. However, all definitions used have been validated by the literature against manual medical record review [29–33]. Next, as this is a secondary data analysis: only those patients who correctly received an ICD or CPT code for their diagnoses and co-morbidities are captured. It is possible that patients with cirrhosis are therefore missed because they were not coded or have not yet been diagnosed with cirrhosis. While the ICD-10 introduced a specific code for NASH in 2015, prior diagnosis for NASH had to be imputed through published algorithms [34,35]. Thus, the reported NASH prevalence prior to 2015 is likely a lower bound of the true NASH prevalence of cirrhosis. We should also note that the NASH cirrhosis terminology was recently replaced by metabolic dysfunction-associated steatotic liver disease (MASLD) on June 24^th^, 2023, but the characteristics of NASH and MASLD are largely similar [74,75]. Fourth, the data source of this study is limited to commercially and Medicare Advantage insured patients. Though, these findings may not apply to uninsured or Medicaid insured patients, approximately 70% of the US population are enrolled in commercial or Medicare Advantage insurance and captures an important proportion of the US population [76]. Data on large cohorts of patients with cirrhosis are limited since large, national data repositories of these patients currently do not exist. Lastly, MELD-Na information, which poses an important aspect for liver transplantation eligibility, were missing in 43% of the patients in our cohort. Because of this, we cannot make strong inferences regarding the MELD-Na, but also highlights the need for timely referral to specialty care (e.g., hepatologists, transplant surgeons) to appropriately manage patient with cirrhosis. Addressing the issues with timely referral and specialist workforce shortage will be paramount in the future care of the patient with cirrhosis [66,73,77]. Nevertheless, our report is important as it highlights the significant and growing public health burden of cirrhosis using one of the largest, longitudinal, cohorts of patients with cirrhosis.

## Conclusions

In conclusion, in this large cohort of insured Americans, the prevalence of cirrhosis in 2018 reached 0.71%, more than a 2.1-fold increase within 6 years. This trend was largely driven by the increase in alcohol-related and NASH cirrhosis. Patients living in the South, West, and those with Medicare Advantage insurance also had disproportionate rises in prevalence, rising by 2.3-, 2.2-, and 1.9-fold respectively over the study period. Cirrhosis represents a growing public health burden and targeted public health interventions to mitigate progression of cirrhosis among high-risk persons (e.g., alcohol-related cirrhosis, NASH, patients with decompensated cirrhosis) need to be considered to curb the observed trend.

## Supporting information

S1 Table Inclusion codes for patients with cirrhosis. (DOCX)

S2 Table Codes cirrhosis complications. (DOCX)

S3 Table S3A Table. Etiology of cirrhosis codes methodology. S3B Table. Non-Mutually Exclusive Etiologies. S3C Table. Definition of NASH and Cryptogenic Cirrhosis. S3D Table. Supporting diagnoses codes for NASH and cohort results.
(DOCX)

S4 Table S4A Table. Model for End Stage Liver Disease (MELD) Score calculation. S4B Table. Definition of Dialysis for calculation in MELD score.
(DOCX)

S5 Table Prevalence (%) by state arranged in ascending order from 2012–2018. (DOCX)

S1 Methods Supplemental material for prevalence calculation. (DOCX)
