# Supplementary material for: Increasing prevalence of cirrhosis among insured adults in the United States, 2012–2018
Source: PLoS One. 2024 Feb 26;19(2):e0298887. doi: 10.1371/journal.pone.0298887 (PMC10896513; doi:10.1371/journal.pone.0298887)
Supplement: S1 Table — (DOCX) [file pone.0298887.s001.docx]

**S1 Table:** Cirrhosis diagnosis codes

| **Cirrhosis diagnosis code** | **ICD-9** | **ICD-10** |
| --- | --- | --- |
| Cirrhosis | 571.2—Alcoholic cirrhosis of the liver  571.5—Cirrhosis of liver without mention of alcohol  571.6—Biliary cirrhosis | K 70.30 Alcoholic cirrhosis of liver  K 70.31 Alcoholic cirrhosis of liver with ascites  K 71.7 cirrhosis (of liver) with toxic liver disease  K74.3 Primary Biliary Cirrhosis  K74.4 Secondary Biliary Cirrhosis  K74.5 Biliary cirrhosis, unspecified  K74.60 Unspecified cirrhosis of liver  K 74.69 Other cirrhosis of the liver  K76.1 cardiac/congestive cirrhosis of liver  P78.81 congenital cirrhosis (of liver)  E83.110 pigmentary cirrhosis (of liver) |
| Varices | 456.0, 456.1, 456.2—Esophageal varices with or without bleeding | i85.00, i85.01 -- Esophageal varices  I86.4 – gastric varices |
| Hepatic Encephalopathy | 572.2—HE  070.0 Viral hepatitis A with hepatic coma  070.2 Viral hepatitis B with hepatic coma  070.22Viral hepatitis B with hepatic coma – chronic, without mention of hepatitis delta  070.23 Viral hepatitis B with hepatic coma – chronic, with hepatitis delta  070.4 Other specified viral hepatitis with hepatic coma  070.41 Acute or unspecified hepatitis C with hepatic coma  070.44 Chronic hepatitis C with hepatic coma  070.49 Other specified viral hepatitis with hepatic coma  070.6 Unspecified viral hepatitis with hepatic coma | B15.0 Viral Hep A with hepatic coma  B16.0 Viral Hep B with delta with hepatic coma  B16.2 Viral Hep B without delta with hepatic coma  B17.11 Acute hepatitis C with hepatic coma  B19.0 Unspecified viral hepatitis with hepatic coma  B19.11 Unspecified Viral Hep B with hepatic coma  B19.21 Unspecified Viral Hep C with hepatic coma |
| Portal Hypertension | 572.3—Portal hypertension | K76.6 Portal hypertension |
| Hepatorenal Syndrome | 572.4—Hepatorenal syndrome | K91.83, K76.7 Hepatorenal syndrome |
| Spontaneous bacterial peritonitis | 567.23—Spontaneous bacterial peritonitis | K65.2 Spontaneous bacterial peritonitis |
| Hepatopulmonary syndrome | 573.5—Hepatopulmonary syndrome | K76.81 Hepatopulmonary syndrome |
