# Supplementary material for: Increasing prevalence of cirrhosis among insured adults in the United States, 2012–2018
Source: PLoS One. 2024 Feb 26;19(2):e0298887. doi: 10.1371/journal.pone.0298887 (PMC10896513; doi:10.1371/journal.pone.0298887)
Supplement: S2 Table — (DOCX) [file pone.0298887.s002.docx]

**S2 Table:** Complications (not mutually exclusive and may be modified after discussion with UHG)

| **Complication Code** | **ICD-9** | **ICD-10** | **CPT code** |
| --- | --- | --- | --- |
| Hepatic encephalopathy | 572.2 HE  070.0 Viral hepatitis A with HE  070.2 Viral hepatitis B with HE  070.22 Chronic HBV and HE  070.23 HBV HDV and HE  070.4 viral hepatitis with HE  070.41 Acute or unspecified HCV with HE  070.44 Chronic HCV with HE  070.49 Other specified viral hepatitis with HE  070.6 Unspecified viral hepatitis with HE | B15.0 Viral Hep A with hepatic coma  B16.0 HBV HDV with HE  B16.2 HBV only with HE  B17.11 acute HCV with HE  B19.0 viral hepatitis with HE  B19.11 HBV with HE  B19.21 Chronic HCV with HE  K71.11 Toxic Liver disease with HE  K72.11 Chronic Hepatic failure with HE | None |
| Ascites^63^ |  | K70.11 Alc hep with ascites  K70.31 etoh cirrhosis with ascites |  |
| Esophageal Varices without bleeding (and no codes at any time for Variceal Bleeding) | 456.1 Esophageal varices without bleeding  456.21 Esophageal varices without bleeding classified elsewhere | i85.00, i85.10 Esophageal varices without bleeding, I86.4 – gastric varices |  |
| Variceal Bleeding | 456.0  ESOPHAGEAL VARICES WITH BLEEDING, 456.20  ESOPHAGEAL VARICES IN DISEASES CLASSIFIED ELSEWHERE WITH BLEEDING | i85.01, i85.11 -- Esophageal varices with bleeding | 43243, 43244 |
| Bacterial Peritonitis | 567.23 Spontaneous bacterial peritonitis | K65.2 Spontaneous bacterial peritonitis |  |
| Hepatocellular Carcinoma | 155.0--Malignant neoplasm of liver, primary | C22.0 Liver cell carcinoma | 79445 |
| Hepatorenal syndrome | 572.4 Hepatorenal syndrome | K91.83, K76.7 Hepatorenal syndrome |  |
| Portal hypertensive | 572.3 Portal hypertension | K76.6 Portal hypertension |  |
| Portal hypertensive complications | 573.5 Hepatopulmonary syndrome | K76.81 Hepatopulmonary syndrome |  |
| TIPS |  |  | 37182 , 37183 TIPS |
| Transplant | 996.82 complications of liver transplant  V42.7 Liver replaced by transplant, 50.59, 50.51 Other transplant of Liver, | Z76.82 Waitlisted, Z48.23 Encounter for aftercare following liver transplant,  T86.4 Complications of liver transplant, T86.40 unspecified, T86.41 rejection, T86.42 failure, T86.43 infection, T86.49 other complication of liver transplant  Z94.4 Liver transplanted | 47133, 47135 Liver allotransplantation; orthotopic, partial or whole, from cadaver or living donor, any age 47140 through 47147 |
