# Supplementary material for: Increasing prevalence of cirrhosis among insured adults in the United States, 2012–2018
Source: PLoS One. 2024 Feb 26;19(2):e0298887. doi: 10.1371/journal.pone.0298887 (PMC10896513; doi:10.1371/journal.pone.0298887)
Supplement: S3 Table — S3A Table. Etiology of cirrhosis codes methodology. S3B Table. Non-Mutually Exclusive Etiologies. S3C Table. Definition of NASH and Cryptogenic Cirrhosis. S3D Table. Supporting diagnoses codes for NASH and cohort results. (DOCX) [file pone.0298887.s003.docx]

**S3A Table:** Etiology of cirrhosis codes methodology

Once patients are included, these codes will define the subgroups of etiologies of cirrhosis for patients in the cohort. Given the clinically informed assumption that cirrhosis is not reversible and these causes usually take years if not decades to cause cirrhosis, we consider a code at any time during the enrollment falling into any subgroups below was contributory to the patients liver disease.

**S3B Table:** Non-Mutually Exclusive etiologies (patients can have more than one etiology for liver disease)

| **Group** | **ICD-9** | **ICD-10** | **CPT** |
| --- | --- | --- | --- |
| Hepatitis B | 070.20, 070.21, 070.22, 070.23, 070.30, 070.31, 070.32, 070.33, V02.61 | B16.0 HBV HDV with HE  B16.1 HBV HDV without HE  B16.2 HBV only with HE  B16.9 acute HBV  B18.0 Chronic HBV with HDV  B18.1 Chronic HBV  B19.9  B19.10 HBV NOS  B19.11 HBV with HE |  |
| Hepatitis C | 070.41, 070.44, 070.51, 070.54, 070.70, 070.71, V02.62 | B17.10 acute HCV  B17.11 acute HCV with HE  B18.2 Chronic HCV  B19.20 HCV NOS  B19.21 Chronic HCV with HE |  |
| Alcohol related | 571.1 acute alcoholic liver disease  571.2 Alcoholic cirrhosis of the liver  571.3 chronic alcoholic liver disease  **Non-liver but EtOH dependence, and/or overdose codes**  291 Alcohol-induced mental disorders  291 Alcohol withdrawal delirium  291.1 Alcohol-induced persisting amnestic disorder  291.2 Alcohol-induced persisting dementia  291.3 Alcohol-induced psychotic disorder with hallucinations  291.4 Idiosyncratic alcohol intoxication  291.5 Alcohol-induced psychotic disorder with delusions  291.8 Other specified alcohol-induced mental disorders  291.81 Alcohol withdrawal  291.82 Alcohol-induced sleep disorder  291.89 Other alcoholic psychosis  291.9 Unspecified alcohol-induced mental disorders  303 Alcohol dependence syndrome  303 Acute alcoholic intoxication  303 Acute alcoholic intoxication in alcoholism unspecified drinking behavior  303.01 Acute alcoholic intoxication in alcoholism continuous drinking behavior  303.02 Acute alcoholic intoxication in alcoholism episodic drinking behavior  303.03 Acute alcoholic intoxication in alcoholism in remission  303.9 Other and unspecified alcohol dependence  303.9 Other and unspecified alcohol dependence unspecified drinking behavior  303.91 Other and unspecified alcohol dependence continuous drinking behavior  303.92 Other and unspecified alcohol dependence episodic drinking behavior  303.93 Other and unspecified alcohol dependence in remission | K70.9 ETOH NOS  K70.10 Alc Hepatitis  K70.11 Alc hep with ascites  K 70.30 Alcoholic cirrhosis of liver  K 70.31 Alcoholic cirrhosis of liver with ascites  **Non-liver but EtOH dependence, and/or overdose codes**  **F10.0-** F10.99 |  |
| Primary biliary cholangitis | 571.6 Biliary cirrhosis | K74.3 Primary Biliary Cirrhosis  K74.4 Secondary Biliary Cirrhosis  K74.5 Biliary cirrhosis, unspecified |  |
| Primary Sclerosing Cholangitis |  | K83.01, K83.09 PSC |  |
| Autoimmune Hepatitis | 571.42 Autoimmune Hepatitis | K75.4 Autoimmune hepatitis |  |
| Cardiac |  | K76.1 cardiac/congestive cirrhosis of liver |  |
| Genetic | 273.4 -- A1AT  275.01, 275.1 –Wilsons  275.03 – hemochromatosis | P78.81 congenital cirrhosis (of liver)  E83.110 pigmentary cirrhosis (of liver)  E83.01 Wilson’s Disease  E88.01 A1AT |  |

**S3C Table:** Definition of NASH and Cryptogenic Cirrhosis and cohort results

| **Group** | **ICD-9** | **ICD-10** | **CPT** |
| --- | --- | --- | --- |
| Presumed NASH based on comorbidities* ^64,65^ | 571.8 + ICD codes for obesity, hypertension, diabetes, and dyslipidemia | K 75.81 Nonalcoholic steatohepatitis  K74.60 + ICD codes for obesity, hypertension, diabetes, and dyslipidemia |  |
| Other/Cryptogenic | Without presence of ICD code that would have otherwise appeared as above | Without presence of ICD code that would have otherwise appeared as above |  |

Non-alcoholic steatohepatitis (NASH) cirrhosis was defined as ICD-10 for NASH cirrhosis or ‘no other etiology’ plus any of the following diagnoses (diabetes, hypertension obesity). ‘No other etiology’ is defined as a patient who does not have any diagnoses codes for hepatitis B (HBV), hepatitis C (HCV), alcohol use disorder (ETOH), biliary, autoimmune, genetic, or cardiac cirrhosis.

| **Definitions** | **Patients with cirrhosis**  **Total =371,482 (100%)** |
| --- | --- |
| No other etiologies | 178,360 (48.01%) |
| No other etiologies and Hypertension | 58,615 (15.78%) |
| No other etiologies and Diabetes | 21,549 (5.80%) |
| No other etiologies and Obesity | 14,441 (3.89%) |
| No other etiologies and either Hypertension or Obesity | 61,673 (16.60%) |
| No other etiologies and either Diabetes or Obesity | 28,758 (7.74%) |
| No other etiologies and either Diabetes or Hypertension | 63,182 (17.01%) |
| No other etiologies and any of Diabetes, Hypertension, or Obesity | 65,337 (17.59%) |
| No other etiologies and any of Diabetes, Hypertension, or Obesity or the ICD 10 code for NASH | 76,189 (20.51%) |

**S3D Table:** Supporting diagnoses codes for NASH

Based on Elixhauser definitions as used in Table 2 from Quan (2005) which has ICD9 and ICD10 codes^1,2^

| **Group** | **ICD-9** | **ICD-10** | **CPT** |
| --- | --- | --- | --- |
| Obesity | 278.0 | E66.x |  |
| Diabetes, and/or diabetic complications | 250.0-250.9  648.0, 775.1 | E10.0-E10.9,  E11.0-E11.9,  E12.0-E12.9,  E13.0-E13.9,  E14.0-E14.9, |  |
| Hypertension and/or hypertensive complications | 401.x,  402.x-405.x,  642.1, 642.2, 642.7, 642.9 | 110.x, 111.x-113.x,115.x |  |

**Refences**

1. Quan H, Sundararajan V, Halfon P, et al. Coding algorithms for defining comorbidities in ICD-9-CM and ICD-10 administrative data. *Medical care*. 2005;43(11):1130-1139. doi:10.1097/01.MLR.0000182534.19832.83

2. Quan H, Li B, Couris CM, et al. Updating and Validating the Charlson Comorbidity Index and Score for Risk Adjustment in Hospital Discharge Abstracts Using Data From 6 Countries. *American Journal of Epidemiology*. 2011;173(6):676-682. doi:10.1093/aje/kwq433
