# Supplementary material for: Increasing prevalence of cirrhosis among insured adults in the United States, 2012–2018
Source: PLoS One. 2024 Feb 26;19(2):e0298887. doi: 10.1371/journal.pone.0298887 (PMC10896513; doi:10.1371/journal.pone.0298887)
Supplement: S4 Table — S4A Table. Model for End Stage Liver Disease (MELD) Score calculation. S4B Table. Definition of Dialysis for calculation in MELD score. (DOCX) [file pone.0298887.s004.docx]

**S4A Table:** Model for End Stage Liver Disease (MELD) Score calculation

| **Laboratory Test Name (allowed range)** | **LOINC codes** |
| --- | --- |
| Platelets | ‘26515-7’,’777-3’,’49497-1’,’778-1’,’74464-9’,’26516-5’,’13056-7’, ‘5907-1 deprecated’, ‘47284-5’, ‘778-1’ |
| AST | ‘30239-8’,’48136-6’, ‘1920-8’ |
| INR (0-20) | ‘34714-6’,’46418-0’, ‘38875-1’, ‘5895-7’, ‘6301-6’, ‘34714-6’, ‘38875-1’, ‘46418-0’, ‘52129-4’, ‘5895-7’, ‘5896-5’, ‘6301-6’, ‘92891-1’ |
| PT | 34528-0 (PT panel), 34528-0 (PT & APTT panel) |
| ALT | '76625-3','1742-6','1743-4','77144-4' |
| Direct Bilirubin | ‘34543-9’ |
| Total Bilirubin (0-60) | '42719-5','59827-6','59828-4','1975-2' |
| Sodium (100-170) | '42570-2','2947-0','32717-1','39792-7','41657-8','39791-9','2951-2','77139-4','51419-0' |
| Creatinine (0-20) | ‘11041-1’, ‘11042-9’, ‘14682-9’, ‘15045-8’, ‘2148-5’, ‘2160-0’, ’35203-9’, '44784-7', ‘51620-3’, ‘51619-5’, ‘38483-4’, ‘59826-8’, ‘21232-4’ |

**S4B Table:** Definition of Dialysis for calculation in MELD score

|  | **ICD-9** | **ICD-10** | **CPT** |
| --- | --- | --- | --- |
| Dialysis | 585.6 ESRD  V45.11 Dialysis status  V56.0  V56.1  V56.2  V56.31 dialysis  V56.32 peritoneal dialysis  V56.8 | Z99.2 Dependence on dialysis  Z49 encounter for dialysis   Z49.0 Preparatory care for renal dialysis   Z49.01 Encounter for fitting and adjustment of extracorporeal dialysis catheter   Z49.02 Encounter for fitting and adjustment of peritoneal dialysis catheter   Z49.3 Encounter for adequacy testing for dialysis   Z49.31 Encounter for adequacy testing for hemodialysis   Z49.32 Encounter for adequacy testing for peritoneal dialysis  N18.6 End stage renal disease | ​90963-90970 |
