# Supplementary material for: Increasing prevalence of cirrhosis among insured adults in the United States, 2012–2018
Source: PLoS One. 2024 Feb 26;19(2):e0298887. doi: 10.1371/journal.pone.0298887 (PMC10896513; doi:10.1371/journal.pone.0298887)
Supplement: S5 Table — (DOCX) [file pone.0298887.s005.docx]

**S5 Table:** Prevalence (%) by state arranged in ascending order from 2012-2018

| **State** | **2012** | **2014** | **2016** | **2018** |
| --- | --- | --- | --- | --- |
| **KS** | 0.19 | 0.25 | 0.28 | 0.31 |
| **AK** | 0.20 | 0.23 | 0.21 | 0.31 |
| **MI** | 0.18 | 0.21 | 0.26 | 0.32 |
| **MT** | 0.19 | 0.29 | 0.34 | 0.32 |
| **MN** | 0.19 | 0.26 | 0.32 | 0.33 |
| **MS** | 0.20 | 0.24 | 0.31 | 0.33 |
| **NE** | 0.20 | 0.22 | 0.30 | 0.35 |
| **IA** | 0.22 | 0.28 | 0.33 | 0.39 |
| **LA** | 0.20 | 0.25 | 0.35 | 0.40 |
| **MD** | 0.23 | 0.32 | 0.39 | 0.43 |
| **WY** | 0.22 | 0.27 | 0.37 | 0.43 |
| **WI** | 0.21 | 0.29 | 0.40 | 0.44 |
| **DE** | 0.27 | 0.35 | 0.45 | 0.44 |
| **VA** | 0.26 | 0.31 | 0.38 | 0.44 |
| **UT** | 0.18 | 0.25 | 0.35 | 0.46 |
| **MA** | 0.28 | 0.40 | 0.40 | 0.47 |
| **WV** | 0.24 | 0.36 | 0.40 | 0.47 |
| **PA** | 0.25 | 0.31 | 0.37 | 0.48 |
| **OH** | 0.24 | 0.32 | 0.40 | 0.49 |
| **IL** | 0.21 | 0.32 | 0.40 | 0.49 |
| **ND** | 0.18 | 0.31 | 0.45 | 0.50 |
| **KY** | 0.22 | 0.28 | 0.38 | 0.50 |
| **MO** | 0.22 | 0.30 | 0.43 | 0.52 |
| **IN** | 0.25 | 0.33 | 0.44 | 0.52 |
| **TN** | 0.31 | 0.39 | 0.48 | 0.54 |
| **NV** | 0.26 | 0.37 | 0.49 | 0.54 |
| **NJ** | 0.26 | 0.37 | 0.47 | 0.54 |
| **NH** | 0.26 | 0.34 | 0.42 | 0.55 |
| **CO** | 0.25 | 0.37 | 0.48 | 0.59 |
| **NY** | 0.31 | 0.42 | 0.52 | 0.60 |
| **AR** | 0.20 | 0.25 | 0.54 | 0.60 |
| **CT** | 0.33 | 0.39 | 0.50 | 0.60 |
| **OK** | 0.28 | 0.41 | 0.52 | 0.62 |
| **FL** | 0.35 | 0.45 | 0.58 | 0.63 |
| **OR** | 0.27 | 0.37 | 0.49 | 0.64 |
| **ME** | 0.25 | 0.34 | 0.44 | 0.64 |
| **NC** | 0.30 | 0.44 | 0.51 | 0.66 |
| **SD** | 0.21 | 0.39 | 0.58 | 0.68 |
| **ID** | 0.31 | 0.49 | 0.62 | 0.68 |
| **AZ** | 0.33 | 0.45 | 0.55 | 0.69 |
| **GA** | 0.26 | 0.28 | 0.63 | 0.71 |
| **CA** | 0.33 | 0.44 | 0.61 | 0.73 |
| **NM** | 0.41 | 0.57 | 0.88 | 0.75 |
| **RI** | 0.47 | 0.59 | 0.70 | 0.78 |
| **SC** | 0.23 | 0.27 | 0.73 | 0.79 |
| **DC** | 0.19 | 0.29 | 0.59 | 0.79 |
| **WA** | 0.35 | 0.52 | 0.80 | 0.82 |
| **TX** | 0.31 | 0.46 | 0.64 | 0.82 |
| **VT** | 0.49 | 0.57 | 0.83 | 0.87 |
| **AL** | 0.38 | 0.46 | 0.60 | 0.90 |
| **HI** | 1.08 | 1.60 | 1.89 | 1.62 |

S5 Table Legend: Prevalence in percentage in ascending order by year and state; AL: Alabama, AK: Alaska, AR: Arkansas, AZ: Arizona, CA: California, CO: Colorado, CT: Connecticut, DC: Washington DC, DE: Delaware, FL: Florida, GA: Georgia, HI: Hawaii, ID: Idaho, IA: Iowa, IL: Illinois, IN: Indiana, KS: Kansas, KY: Kentucky, LA: Louisiana, ME: Maine, MA: Massachusetts, MD: Maryland, MI: Michigan, MN: Minnesota, MO: Missouri, MS: Mississippi, MT: Montana, NC: North Carolina, NE: Nebraska, NH: New Hampshire, NJ: New Jersey, NM: New Mexico, NV: Nevada, NY: New York, ND: North Dakota, OH: Ohio, OK: Oklahoma, OR: Oregon, PA: Pennsylvania, RI: Rhode Island, SC: South Carolina, SD: South Dakota, TN: Tennessee, TX: Texas, UT: Utah, VA: Virginia, VT: Vermont, WA: Washington, WI: Wisconsin, WV: West Virginia, WY: Wyoming
