# Supplementary material for: Increasing prevalence of cirrhosis among insured adults in the United States, 2012–2018
Source: PLoS One. 2024 Feb 26;19(2):e0298887. doi: 10.1371/journal.pone.0298887 (PMC10896513; doi:10.1371/journal.pone.0298887)
Supplement: S1 Methods — (DOCX) [file pone.0298887.s006.docx]

**S6** **Methods:** Supplemental material for prevalence calculation

**Dealing with Observational Gaps**

Patients that have gaps in their UHG enrollment may or may not have been diagnosed with cirrhosis in that year. Similarly, when patients first enroll in UHG data, we do not know if they have been previously diagnosed with cirrhosis. We only observe the first cirrhosis-related event - which possibly occurs much later than UHG enrollment. We deal with this missing data problem by assuming that patients with cirrhosis have cirrhosis-related events at least every k years. We then use observed events to mark if patients could have had cirrhosis in years following a gap or initial enrollment.

Formally, let t denote a calendar year (ending in July 15), where t = 1 (2011) denotes our first year of data and t = T the final year (2018).

We use notation for sets of patients:

N(t) is the set of all patients enrolled in UHG at year t and are older than 18 in year t.

NC(t) is the subset of UHG patients who have been diagnosed with cirrhosis before or in year t.

The absolute value sign is used to denote the number of individuals in each set. Our goal is to measure |NC(t)|/|N(t)|, the prevalence of cirrhosis at year t.

The UHG Masterfile of all insured patients provides |N(t)|.  Difficulties arise in estimating |NC(t)| because we do not know whether the first cirrhosis event for an individual in our data is their diagnosis or a follow up visit. We can bound |NC(t)| using the subset of UHG patients that have cirrhosis ICD codes (the dataset we are working with). We define useful notation for subgroups of patients.

NC^*^(t) is the set of patients whose record has a cirrhosis event in some year before or during t, and who are insured by UHG in year t.

NC_k_(t) for k > 1 is the set of patients insured by UHG in year t whose record has no ICD code for cirrhosis up to year t, did not appear in UHG for at least one year t – c for any c such that 0 < c ≤ k - 1, and also whose record has a cirrhosis event in some year after t and up to t – c + k. In English, this is the set of patients for whom it is possible they had a cirrhosis event while not enrolled with UHG, did not have a cirrhosis event in year t, and had their first UHG cirrhosis event no more than k years after the most recent possible cirrhosis event while not enrolled in UHG.

The value k is interpreted as the largest number of years a patient will go between cirrhosis-related events for patients that have been diagnosed with cirrhosis, with k = 1 meaning a visit every year (no gaps), k = 2 a visit every two years (a possible gap of one year), etc. NC_k_(t) represents the maximal number of UHG enrollees who could have cirrhosis given the assumption of k but have not had cirrhosis recorded in the data by year t.

To define NC_k_(t) for k = 1, note that k = 1 implies that patients have a cirrhosis event every year, there could not be gaps between events. Under the assumption k = 1, if a patient is in the UHG data and has not yet had a cirrhosis event, it must be that they do not have cirrhosis, thus NC_k_(t) = 0 for k = 1.

**Bounds**

It must be that NC^*^(t) is a subset of NC(t) (the patients for whom we observe cirrhosis event during or prior to year t must be a subset of the patients that actually have been diagnosed with cirrhosis). This gives a lower bound on |NC(t)|, namely |NC^*^(t)| ≤ |NC(t)|. This is the lower bound reported in prevalence tables.

If patients have cirrhosis events at least every k years, then |NC^*^(t)| +|NC_k_(t)| ≥ |NC(t)|. In words, all individuals who have been diagnosed must be either (1) those who have had had a cirrhosis event recorded in UHG during or prior to t or (2) patients who could have cirrhosis if diagnosed while outside of UHG with possible gaps of k years. Note for k > 1, that group (2) will likely include some patients who do not have cirrhosis. Thus, |NC^*^(t)| +|NC_k_(t)| is the upper bound reported.

For k = 1, NC_k_(t) = 0, and the lower and upper bounds coincide.

**Choice of k**

For our primary analysis we use k = 2 to create upper bounds, where k is defined as years. We also report estimated upper bounds with k = 1 and k = 3. The choice of k = 2 is informed by observations made using UHG data. In addition, we used an external data set, Chicago Area Patient-Centered Outcomes Research Network (CAPriCORN), for which we observe all health

system visits for patients in the Chicago metropolitan area.^17^ Similar observations were made with CAPriCORN. Therefore, in order not to miss those that enter UHG and have cirrhosis, but do not have a clinical visit within 1 or 2 years, we choose (K-1) to impute those patients for year 2018.

**Extrapolation in 2018**

Our use of k as described above is motivated by the fact that patients may be diagnosed prior to observing a cirrhosis event in the administrative dataset. This is a particular issue with data from the final year of data (2018), as we do not observe cirrhosis events in future years for which the methodology and use of k allows us to create upper bounds. This is also an issue when k = 3 in 2017, and when k = 2 and k = 3 in 2018. To this end, we extrapolate the upper bounds using the rate of new cirrhosis patients that are affected by the k methodology in 2015.  Highlighted in blue are the adjusted numbers, accounting for the fact that some patients with diagnosed cirrhosis are in the cohort before the diagnosis is captured by a patient visit. k equals the years of enrollment accounted for before diagnosis of cirrhosis occurs. For example, in 2015 there were 4,800,048 new patients in the dataset. Of these, 7,868 did not have a cirrhosis diagnosis captured in 2015 but had a visit with the cirrhosis diagnosis in 2016. They were marked in the upper bound when using k = 2. The percentage of newly diagnosed patients with cirrhosis in year 2 of enrollment is **0.1639%**. In 2018, there were 5,132,366 new patients in the cohort for k=2, **0.1639%** were added, namely 8,413 patients (see highlighted in blue). The same approach was used to calculate k=3. As most patients were enrolled two but not three years, k=2 was used for the calculation of prevalence. More details are available from the authors.

Patients with cirrhosis by k

| **Year** | **NA(t)** | **Lower Bound** | **Upper Bound k = 1** | **Upper Bound k = 2** | **Upper Bound**  **k = 3** | **N(t)** |
| --- | --- | --- | --- | --- | --- | --- |
| **2011** | 39,258 | 39,258 | 39,258 | 74,377 | 98,905 | 19,012,698 |
| **2012** | 59,922 | 59,922 | 59,922 | 67,025 | 95,499 | 19,740,634 |
| **2013** | 76,485 | 76,485 | 76,485 | 84,204 | 93,018 | 20,653,786 |
| **2014** | 83,153 | 83,153 | 83,153 | 89,954 | 98,872 | 19,618,517 |
| **2015** | 91,150 | 91,150 | 91,150 | 99,018 | 107,779 | 19,036,572 |
| **2016** | 110,752 | 110,752 | 110,752 | 122,385 | 133,670 | 20,202,190 |
| **2017** | 129,815 | 129,815 | 129,815 | 140,755 | 159,380 | 21,771,537 |
| **2018** | 140,804 | 140,804 | 140,804 | 149,217 | 161,658 | 21,110,112 |

Prevalence of cirrhosis by k

| **Year** | **Lower Bound** | **Upper Bound k = 1** | **Upper Bound k = 2** | **Upper Bound k = 3** |
| --- | --- | --- | --- | --- |
| **2011** | 0.0021 | 0.0021 | 0.0039 | 0.0052 |
| **2012** | 0.003 | 0.003 | 0.0034 | 0.0048 |
| **2013** | 0.0037 | 0.0037 | 0.0041 | 0.0045 |
| **2014** | 0.0042 | 0.0042 | 0.0046 | 0.005 |
| **2015** | 0.0048 | 0.0048 | 0.0052 | 0.0057 |
| **2016** | 0.0055 | 0.0055 | 0.0061 | 0.0066 |
| **2017** | 0.006 | 0.006 | 0.0065 | 0.0073 |
| **2018** | 0.0067 | 0.0067 | 0.0071 | 0.0077 |
